# The long-term effect of screening and lifestyle counseling on changes in physical activity and diet: the Inter99 Study – a randomized controlled trial

**DOI:** 10.1186/s12966-015-0195-3

**Published:** 2015-03-06

**Authors:** Sophie Baumann, Ulla Toft, Mette Aadahl, Torben Jørgensen, Charlotta Pisinger

**Affiliations:** University Medicine Greifswald, Institute of Social Medicine and Prevention, Walther-Rathenau-Str. 48, DE-17475 Greifswald, Germany; Research Centre for Prevention and Health, Building 84/85, Glostrup Hospital, Ndr, Ringvej, DK-2600 Glostrup Denmark; Faculty of Health Science, University of Copenhagen, Copenhagen, Denmark; Faculty of Medicine, University of Aalborg, Aalborg, Denmark

**Keywords:** Inter99, Lifestyle intervention, Population level, Multi-behavior, Maintenance

## Abstract

**Background:**

Multi-factorial intervention studies have been found to be successful in the initiation of lifestyle changes. However, little is known about the longer-term maintenance of health behavior improvements. The purpose of this study was to investigate whether improvements in physical activity and dietary habits achieved in a population-based multi-factorial lifestyle intervention of five years duration were maintained five years after intervention activities have ended.

**Methods:**

The study was a population-based randomized controlled trial, Inter99 (1999–2006), Copenhagen, Denmark. Over five years, all participants in the intervention group (*n* = 6,091) received individual lifestyle counseling; participants at high risk of ischemic heart disease – according to pre-specified criteria – were also offered group-based counseling. The control group (*n* = 3,324) was followed by questionnaires. Both groups were followed one, three, five, and ten years after baseline. Changes in physical activity and dietary habits (intake of vegetables, fruit, fish, and saturated fat) during and after the intervention were investigated using random-coefficient models.

**Results:**

Five years after the intervention, women in the intervention group reported greater improvements in the intake of fruit (*M*_Δ_ = 90.2 g/week, *p* = 0.041) and less intake of saturated fat (OR = 0.30, 95% CI: 0.17–0.54) than the control group. Men in the intervention group reported greater improvements in physical activity (*M*_Δ_ = 19.6 min/week, *p* = 0.003) and less intake of saturated fat (OR = 0.31, 95% CI: 0.17–0.56) than the control group. Improvements in the intake of vegetables and fish achieved during the intervention were not maintained in the longer-term.

**Conclusions:**

Screening and lifestyle counseling had sustained effects on physical activity and dietary habits five years after its discontinuation. The patterns of long-term changes in lifestyle differed across behaviors and between men and women.

**Trial registration:**

ClinicalTrials.gov (NCT00289237)

## Background

Unhealthy lifestyle behaviors such as unhealthy diet and physical inactivity have been identified as major risk factors for several health outcomes including cardiovascular diseases, diabetes, obesity, and cancer [1]; 10% of global disability-adjusted life years can be attributed to unhealthy diet and physical inactivity [2]. The opportunity to greatly reduce premature deaths and the burden of disease by improvements in lifestyle [3] has led to increasing research efforts to identify the most effective strategies. On the individual level, it is important not only to investigate the initiation and short-term lifestyle behavior changes, but also the maintenance of a healthy lifestyle.

Maintenance of lifestyle changes is crucial for substantial effects on public health. Indeed, only few multi-factorial intervention trials assessed lifestyle factors over more than twelve months, and fewer measured them after discontinuation of the intervention [4]. Although many studies were successful in the initiation of lifestyle changes, improvements tended to decline in the longer-term or once the intervention is completed [4-7]. Generally, it appears that healthy dietary habits are somewhat better maintained than physical activity [8-10]; some important large-scale and long-term trials showed sustained effects on diet [11-13]. One diabetes care trial was successful in producing sustained improvements in both diet and physical activity [14]. However, most of the trials involved intensive, expensive, and demanding intervention programs and/ or included groups of highly selected persons, which may limit their public health impact. Much remains to be learned about how to effectively produce sustained and comprehensive lifestyle changes on a population level.

The Inter99 study is a multi-factorial intervention study to prevent ischemic heart disease (IHD) in the general population by screening and individualized comprehensive lifestyle counseling. Previously, we have shown improvements in physical activity and dietary habits during the intervention period [15-17]. The question now is whether the effects lasted after its discontinuation. The aim of this study was to investigate whether improvements in physical activity and dietary habits achieved in a population-based lifestyle intervention of five years duration were maintained five years after intervention activities ended.

## Methods

### Study population

Inter99 is a population-based randomized intervention study initiated in March 1999 and completed in April 2006 and conducted at the Research Centre for Prevention and Health, The Capital Region, Denmark. The aim of the study was to prevent IHD (and other lifestyle-related diseases) by non-pharmacological intervention focusing on comprehensive improvements in lifestyle. Informed written consent was obtained from all participants. The Inter99 study was approved by the Copenhagen County Ethical Committee (KA 98155) and the National Board of Health.

**Figure 1:**
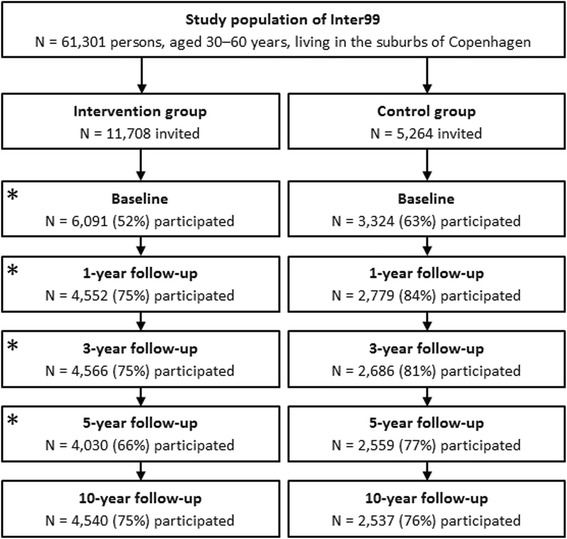
**Flow of participants.** *Note*: Asterisk (*) indicates individual lifestyle counseling and group-based counseling offered to all persons at high risk.

As described elsewhere [18], the study population comprised all 61,301 persons born in 1939–40, 1944–45, 1949–50, 1954–55, 1959–60, 1964–65, and 1969–70 living in the south-western part of Copenhagen County (Figure 1). An age- and sex-stratified random sample of 13,016 persons was drawn from the study population from the Civil Registration System and apriori randomized into two groups comprising 90% (group A: high-intensity intervention, *n* = 11,708) and 10% (group B: low-intensity intervention, *n* = 1,308). The persons randomized to group A and B were invited for a health examination, risk assessment, and individual lifestyle counseling via ordinary mail. The invitation included a questionnaire to be completed in advance. Those not responding received up to two reminder mails. Participants did not receive any incentives. From the remaining 48,285 persons in the study population, a random sample of 5,264 persons equally distributed on sex and age was drawn and followed by questionnaires (group C – control group).

Of the 13,016 invited persons in intervention group A and B, 82 were non-eligible as they had died or were not traceable, leaving 12,934 for invitation. A total of 6,906 (53.4%) turned up for the investigation at the centre. Of these, 122 were excluded due to alcoholism, drug abuse, or linguistic barriers, leaving 6,784 (52.5%). In group C, a total of 3,324 (63.1%) returned the questionnaire. The participation rate was lower than expected resulting in a too small group B and therefore a high risk of type II error. Power calculations before study start were based on an expected participation rate of 70% (for details: [18]). Thus, this paper only includes the 6,091 participants from the intervention group A (henceforth referred to as ‘intervention group’) and the 3,324 participants from group C (henceforth referred to as ‘control group’).

### Intervention

The intervention is described in detail elsewhere [16-19]. At baseline, all participants completed self-report questionnaires and underwent a health examination and an assessment of their individual cardiovascular risk using the Copenhagen Risk Score [20]. They were classified as being at high-risk if they had either an absolute risk in the upper quintile of the distribution stratified according to sex and age, or if they had one or more of the following risk factors: systolic blood pressure ≥ 160 mmHg and/or in antihypertensive treatment, total cholesterol ≥ 7.5 mmol/l and/or treated with statins, body mass index ≥ 30 kg/m^2^, glucose intolerance, diabetes, or daily smoking.

Based on their lifestyle and personal cardiovascular risk score, each participant received a tailored individual lifestyle counseling focusing on smoking, physical activity, diet, and/or alcohol consumption; regardless of whether classified as being at high or low risk. The main goals were: non-smoking; moderate physical activity four hours per week or more; decrease in saturated fat; substitution of saturated fat with unsaturated fat; increase in intake of fruit, vegetables, and fish; and less than 14/ 21 alcoholic drinks per week for women/ men according to national guidelines at that time. Those who already met recommendations were supported to maintain their healthy lifestyle. The counseling sessions lasted 15–45 minutes and were conducted by a nurse, dietitian, or doctor trained in motivational interviewing [21]. The intervention was based on elements of the Health Belief Model [22], the Social Cognitive Theory [23], and the Transtheoretical Model [24].

Besides, high-risk persons in the intervention group were offered group-based counseling on smoking cessation or on diet and physical activity, depending on their lifestyle and motivation to change. Over four to six months, the groups met six times for two hours each time. The sessions on physical activity and diet were led by a nurse or dietitian. At baseline, 47% of those who were offered group-based diet and physical activity counseling [25] and 27% of those who were offered group-based smoking cessation counseling accepted participation [26].

### Follow-up

All high-risk persons in the intervention group were re-invited one and three years after baseline for a health examination, completion of a questionnaire, risk assessment, and individual and group-based lifestyle counseling. Low-risk participants were followed by questionnaire. Five years after baseline, all participants eligible at baseline were re-invited for a final screening, individual counseling, and a plan for maintenance. The control group was followed by questionnaire only. Ten years after baseline, all participants eligible at baseline received post-intervention follow-up questionnaires. At all follow-ups, persons not responding received up to two reminder letters. Participants did not receive any incentives.

### Measures

#### Behavioral outcomes

Self-administrated questionnaires including questions about physical activity and dietary habits were obtained at baseline, one-, three-, five-, and ten-year follow-up. *Physical activity* during leisure time was assessed using two questions: (1) “In your leisure time, how many hours a week are you physically active?” (0 min; 1 h/week; approx. 2–3 h/week; approx. 4–6 h/week; and ≥ 7 h/week) and (2) “How much time do you spend walking, cycling, or running on your way to and from work?” (<15 min; 15–30 min; 30 min–1 h; ≥ 1 h; and I do not work at the moment). The level of physical activity was calculated by summing responses to the two questions converted into minutes per week using a five-day working week.

Dietary habits were measured using a 48-item food frequency questionnaire (FQQ) including nine categories of questions: Number of meals per day (1); type of bread (2); type of spread used on bread (3); cheese, meat, fish, etc. laid on bread (4); hot meals (5); type of fat used for cooking (6); accompaniments to hot meals (7); vegetables (8); and fruits (9). In category 2, 3, and 6, participants were asked to report the type most often used, with the possibility to choose more than one type. In category 4, 5, 7, and 8, participants were asked to report the frequency of eating different kinds of food items in the particular category in the last week (0; 1–2; 3–4; or 5–7 times a week). Dietary outcomes included *intake of saturated fat* (saturated fat for cooking and on bread: yes/ no), *fruits* (0; 1; 2; 3; 4; 5; 6; >6 servings a day), *vegetables* (g/week), and *fish* (g/week)*.* The intake of fish and vegetables was calculated by multiplying the frequency of consuming fish for lunch, fish for dinner, salads, and cooked vegetables with standard portion sizes [27]; the intake of fish and vegetables was summarized. The FFQ has been validated against a 28-day diet history and biomarkers (for further details: [28]).

#### Baseline covariates

A self-administrative questionnaire including questions about socio-demographic and health-related variables was obtained at baseline. Socio-demographic variables included *sex*, *age* (30, 35, 40, 45, 50, 55, and 60 years), *living with a partner* (being married or cohabiting: yes/no), *education* (none; <1; 2–4; and ≥4 years of vocational training/higher education) and *employment* status (employed/unemployed). *Self-perceived health* was recorded in five categories (excellent; very good; good; fair; and poor); due to few observations in the extreme response categories, excellent and poor were merged with very good and fair, respectively. *Smoking* was recorded in four categories (smoker; ex-smoker; occasional smoker; and daily smoker). *Alcohol consumption* was based on average weekly consumption of alcoholic beverages and categorized in five groups (0; 1–7; 8–14; 15–21; and >21 units of ethanol per week; with 1 unit = 15 cl/ 12 g). *Physical activity during leisure time* was assessed by asking the participants to categorizing their leisure time physical activity level in one of four categories (mainly sedentary; moderate activity; regular exercise; and heavy training) [29]. *Being limited in stair-climbing* was measured as being limited in climbing several flights of stairs because of one’s health (yes/no). Based on the FFQ and Danish Dietary Guidelines [30-32], the *Dietary Quality Score* was calculated as an indicator of the overall dietary quality using three categories (healthy; average; and unhealthy dietary habits). The development and validation of the score has been described in more detail previously [33]. Further, participants’ *self-perceived risk of IHD associated with their diet* (yes/ no/ do not know) was assessed.

### Statistical analyses

Data were analyzed using STATA/SE 13.1 [34]. The control group included a larger proportion of persons in the lower and upper age group at baseline than the intervention group due to differences in sampling method [18]. Thus, analyses comparing baseline characteristics between study groups included age-adjusted Cochran-Mantel-Haenszel tests and age-adjusted linear regression models. Multiple logistic regression analyses were used for comparing participants and non-participants at follow-ups.

Piecewise random-coefficient models including a random intercept and two random slopes (active and post-intervention phase) were estimated to determine the effects of the intervention on physical activity and dietary habits over time. All analyses were stratified on sex. Outcomes were changes in physical activity (min/week) and the intake of saturated fat (use = 1/no use = 0), vegetables (g/week), fruit (g/week), and fish (g/week) from baseline to ten years. Net changes defined as intervention minus control group changes from baseline to follow-up and effect sizes (Cohen’s *d* [35]) were calculated.

A maximum likelihood estimator with robust standard errors was used; numerical integration was carried out for models including binary variables. Thus, all models were estimated under a missing at random assumption [36] using all available data from participants with baseline responses. Results were adjusted for baseline variables that were either differently distributed between study groups at baseline or between participants and non-participants at follow-ups: age, smoking, alcohol consumption, education, self-rated health, employment, and living with a partner. Further, models for physical activity included dietary quality and being limited in climbing stairs as covariates; models for dietary habits included physical activity and self-rated IHD risk associated with diet.

## Results

### Study sample

Baseline sample characteristics are shown in Table 1. Men in the intervention group were less often smokers and more often perceived a risk of IHD associated with diet compared to the control group (*p*s < 0.01). Women in the intervention group were more often employed and reported a higher level of subjective health compared to the control group (*p*s < 0.001). No significant group differences in outcome variables at baseline were found among men, whereas women in the control group had a lower intake of fish (*p* < 0.001).

**Table 1:** **Baseline sample characteristics stratified by sex and study group**

	**Men**		**Women**	
	**Intervention**	**Control**	**Intervention**	**Control**
	***N*** **= 2,965**	***N*** **= 1,568**	***N*** **= 3,126**	***N*** **= 1,756**
**Socio-demographic variables**				
Age, *n* (%)				
*30–35 years*	415 (14.0)	365 (23.3)	514 (16.4)	478 (27.2)
*40–50 years*	1,822 (61.4)	678 (43.2)	1,908 (61.0)	774 (44.1)
*55–60 years*	728 (24.6)	525 (33.5)	704 (22.5)	504 (28.7)
Living with a partner, *n* (%)				
*Yes*	2,481 (85.0)	1,300 (84.6)	2,468 (80.2)	1,388 (80.7)
Education, *n* (%)				
*None*	450 (16.6)	245 (17.6)	535 (18.7)	335 (20.9)
*≤1 year*	65 (2.4)	41 (2.9)	199 (6.9)	104 (6.5)
*2–4 years*	1,759 (65.2)	900 (64.5)	1,911 (66.6)	1,036 (64.6)
*5–9 years*	427 (15.8)	209 (15.0)	223 (7.8)	128 (8.0)
Employed, *n* (%)				
*Yes*	2,609 (89.8)	1,343 (86.6)	2,618 (85.4)	1,390 (80.1)
**Health-related variables**				
Self-perceived health, *n* (%)				
*Excellent/ very good*	1,008 (34.4)	585 (37.9)	979 (31.5)	630 (36.3)
*Good*	1,637 (56.0)	829 (53.6)	1,760 (56.7)	891 (51.4)
*Fair/ poor*	281 (9.6)	132 (8.5)	365 (11.8)	213 (12.3)
Daily smoking, *n* (%)				
*Yes*	1,083 (36.9)	643 (41.4)	1,085 (35.0)	633 (36.3)
Alcohol use (drinks/week), *n* (%)				
*0*	222 (7.8)	112 (7.4)	410 (14.0)	291 (17.5)
*1–7*	980 (34.4)	563 (37.1)	1,610 (54.9)	863 (51.9)
*8–14*	651 (22.8)	332 (21.9)	566 (19.3)	317 (19.1)
*15–21*	426 (15.0)	187 (12.3)	215 (7.3)	128 (7.7)
*>21*	571 (20.0)	323 (21.3)	130 (4.5)	64 (3.8)
Physical activity, *n* (%)				
*Mainly sedentary*	642 (22.3)	355 (23.4)	664 (21.7)	391 (23.1)
*Moderate activity*	1,624 (56.3)	863 (56.8)	2,026 (66.2)	1,076 (63.5)
*Regular exercise/ heavy training*	617 (21.4)	300 (19.8)	371 (12.1)	228 (13.4)
Limited in stair-climbing, *n* (%)				
*Yes*	299 (10.3)	201 (13.1)	547 (17.8)	349 (20.1)
Dietary quality, *n* (%)				
*Healthy*	282 (9.9)	118 (8.0)	546 (18.0)	257 (15.6)
*Average*	1,979 (69.8)	1,004 (68.5)	2,142 (70.7)	1,138 (69.1)
*Unhealthy*	576 (20.3)	345 (23.5)	342 (11.3)	253 (15.3)
Self-rated IHD risk of diet, *n* (%)				
*Yes*	433 (15.0)	201 (13.2)	328 (10.8)	183 (10.7)
**Outcome variables at baseline**				
Intake of saturated fat^a^, *n* (%)				
*Yes*	2,599 (88.7)	1,340 (86.6)	2,654 (85.6)	1,454 (83.6)
Intake of vegetables (g/week), *M* ± *SD*	415.3 ± 255.4	401.8 ± 251.8	476.2 ± 263.0	464.0 ± 265.3
Intake of fruit (g/week), *M* ± *SD*	623.1 ± 775.8	622.1 ± 772.5	932.3 ± 889.4	935.8 ± 929.8
Intake of fish (g/week), *M* ± *SD*	158.0 ± 120.0	163.6 ± 128.0	159.4 ± 122.2	146.5 ± 125.2
Physical activity (min/week), *M* ± *SD*	285.9 ± 163.2	298.3 ± 162.4	290.9 ± 161.4	286.9 ± 165.0

*Note*: *M* = mean, *SD* = standard deviation; ^a^for cooking and on bread.

With regard to loss to follow-up, a lower level of physical activity at baseline was predictive for non-response at ten-year follow-up among women (*p* < 0.01). Among men, a higher baseline intake of fish was predictive for non-response at one-, three-, and five-year follow-up (*ps* < 0.05), a higher baseline intake of vegetables at one-year follow-up (*p* < 0.05), and a lower baseline level of physical activity at one- (*p* < 0.01), five-, and ten-year follow-up (*p* < 0.05).

### Lifestyle changes during and after intervention

#### Physical activity

**Figure 2:**
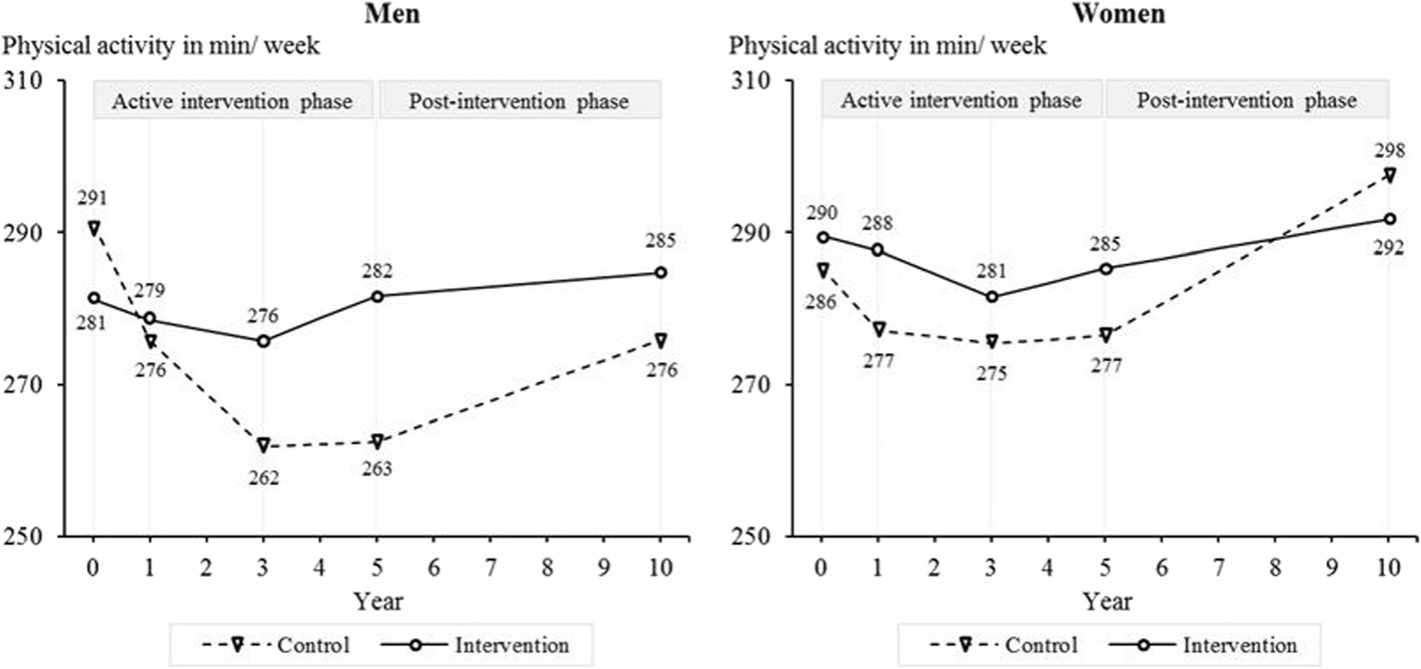
**Level of physical activity among women and men from baseline to 10-year follow up.** *Note*: Results are adjusted for baseline age, living with a partner, education, employment, diet, alcohol consumption, smoking, self-rated health, and being limited in climbing stairs. Year 0 = baseline.

Men in the intervention group decreased their physical activity less through the five years of intervention (net change [*M*_Δ_] = 29.0 min/week, 95% CI: 16.8–41.3, *p* < 0.001, Cohen’s *d* = 0.47) and five years after its discontinuation (*M*_Δ_ = 18.8 min/week, 95% CI: 5.7–31.9, *p* = 0.005, *d* = 0.32) than the control group (Figure 2). For women, no significant differences between groups were found (5-year follow-up: *M*_Δ_ = 4.8 min/week, 95% CI: -6.9–16.5, *p* = 0.425, *d* = 0.06; 10-year follow-up: *M*_Δ_ = -9.8 min/week, 95% CI: -22.0–2.4, *p* = 0.114, *d* = -0.18).

#### Dietary habits

**Figure 3:**
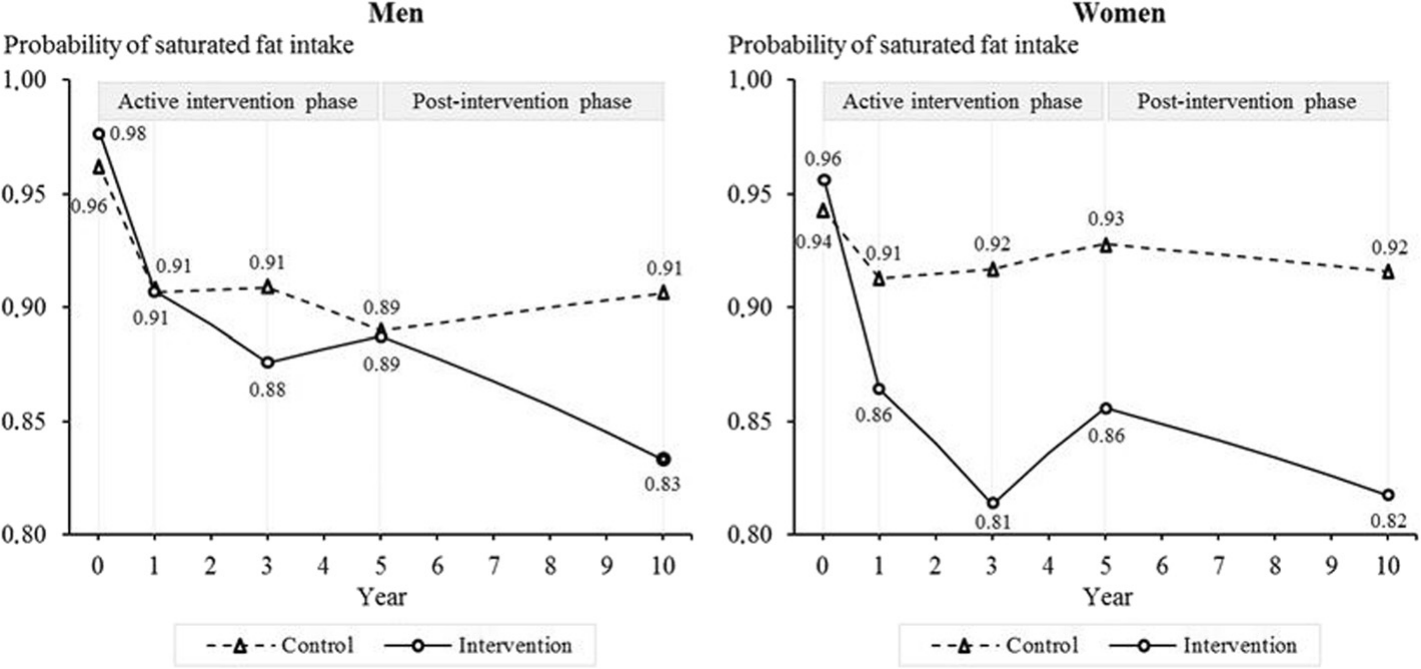
**Intake of saturated fat among women and men from baseline to 10-year follow-up.** *Note*: Results are adjusted for baseline age, living with a partner, education, employment, physical activity, alcohol consumption, smoking, self-rated health, and self-rated risk of IHD associated with dietary habits. Year 0 = baseline.

Compared to the control group, men and women in the intervention group reported a larger decrease in their intake of saturated fat through the five years of intervention (men: OR = 0.55, 95% CI: 0.34–0.90, *p* = 0.019, *d* = -0.24; women: OR = 0.34, 95% CI: 0.22–0.52, *p* < 0.001, *d* = -0.48) and five years after its discontinuation (men: OR = 0.29, 95% CI: 0.16–0.54, *p* < 0.001, *d* = -0.45; women: OR = 0.30, 95% CI: 0.17–0.54, *p* < 0.001, *d* = -0.51) (Figure 3).

**Figure 4:**
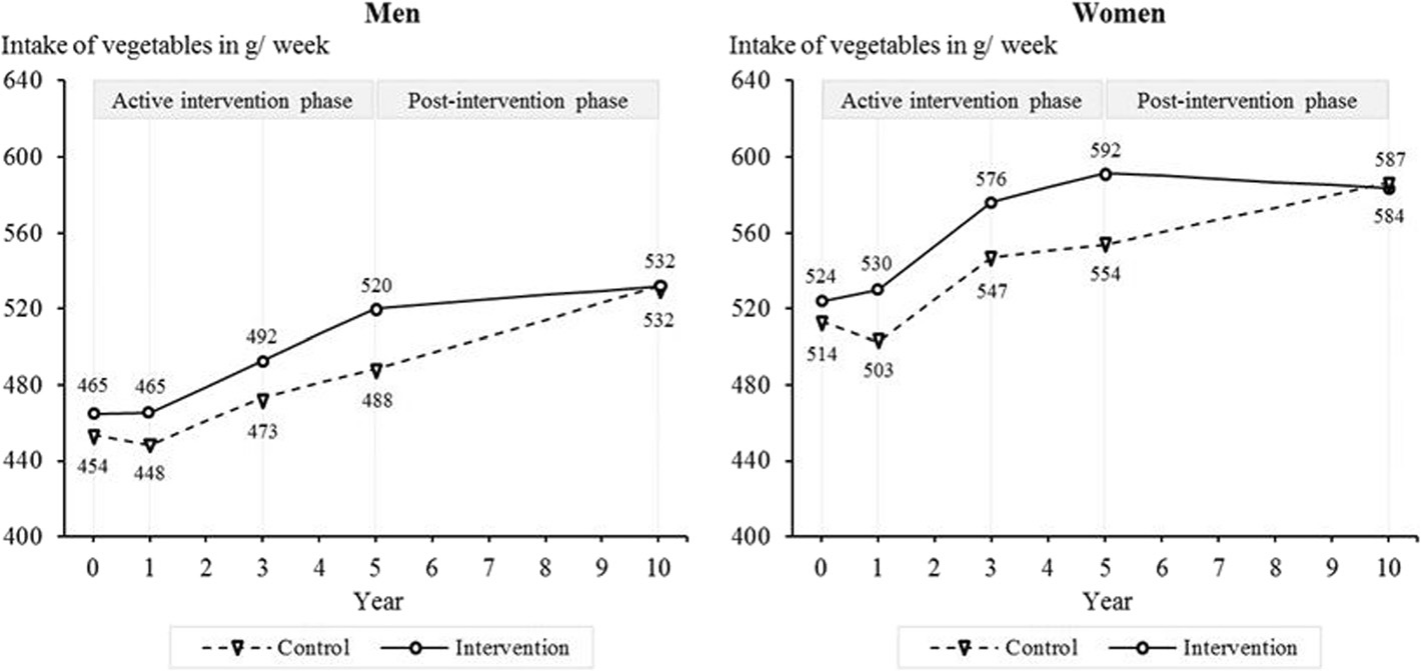
**Intake of vegetables among women and men from baseline to 10-year follow-up.** *Note:* Results are adjusted for baseline age, living with a partner, education, employment, physical activity, alcohol consumption, smoking, self-rated health, and self-rated risk of IHD associated with dietary habits. Year 0 = baseline.

With regard to the intake of vegetables (Figure 4), improvements in the intervention group compared to the control group were greatest at five years (men: *M*_Δ_ = 20.7 g/week, 95% CI: -1.0–42.4, *p* = 0.062, *d* = 0.16; women: *M*_Δ_ = 27.8 g/week, 95% CI: 6.5–49.1, *p* = 0.010, *d* = 0.32). At ten-year follow-up, the two groups did not differ significantly regarding the intake of vegetables (men: *M*_Δ_ = -11.3 g/week, 95% CI: -33.6–11.0, *p* = 0.321, *d* = -0.12; women: *M*_Δ_ = -13.0 g/week, 95% CI: -34.9–9.0, *p* = 0.247, *d* = -0.04).

**Figure 5:**
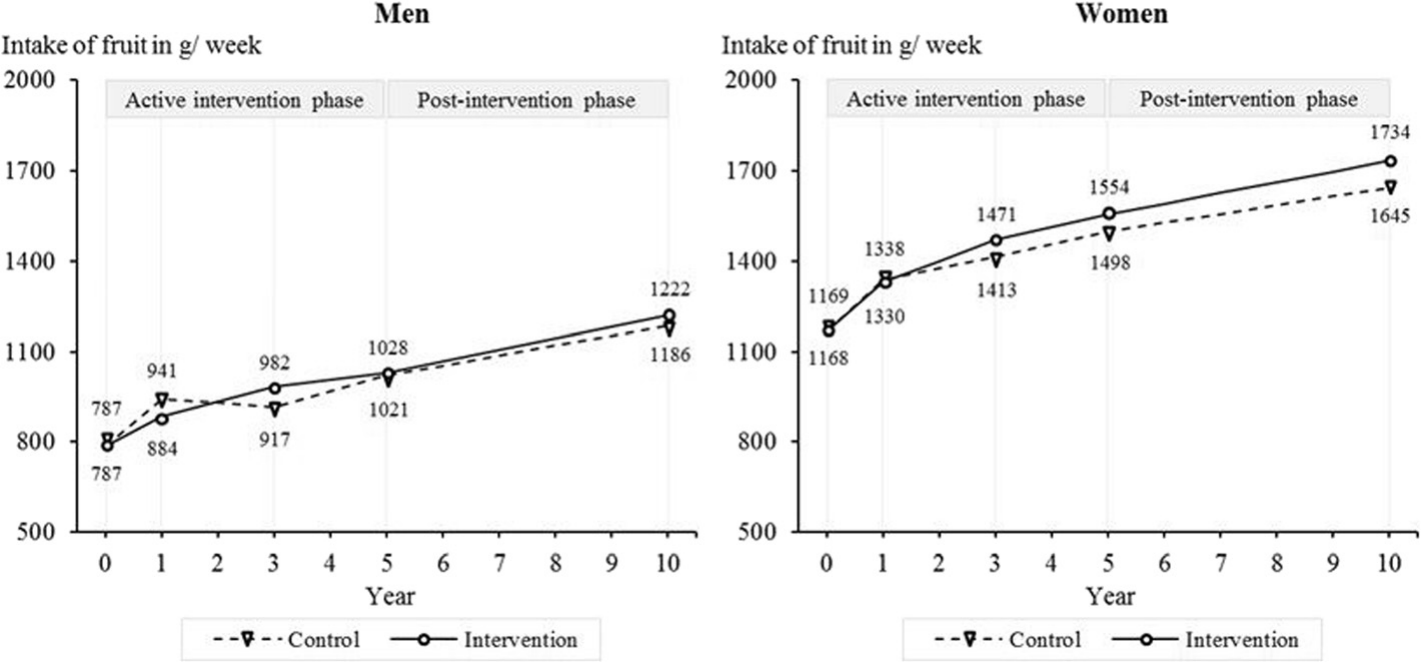
**Intake of fruits among women and men from baseline to 10-year follow-up.** *Note:* Results are adjusted for baseline age, living with a partner, education, employment, physical activity, alcohol consumption, smoking, self-rated health, and self-rated risk of IHD associated with dietary habits. Year 0 = baseline.

Men in the intervention and control group did not differ significantly regarding their intake of fruit at five years and beyond (10-year follow-up: *M*_Δ_ = 46.1 g/week, 95% CI: -36.8–129.0, *p* = 0.115, *d* = 0.06) (Figure 5). Among women, the effect of the intervention on fruit intake increased over time. At ten-year follow-up, women in the intervention group reported a larger increase in their fruit intake than women in the control group (*M*_Δ_ = 90.2 g/week, 95% CI: 3.6–276.9, *p* = 0.041, *d* = 0.15).

**Figure 6:**
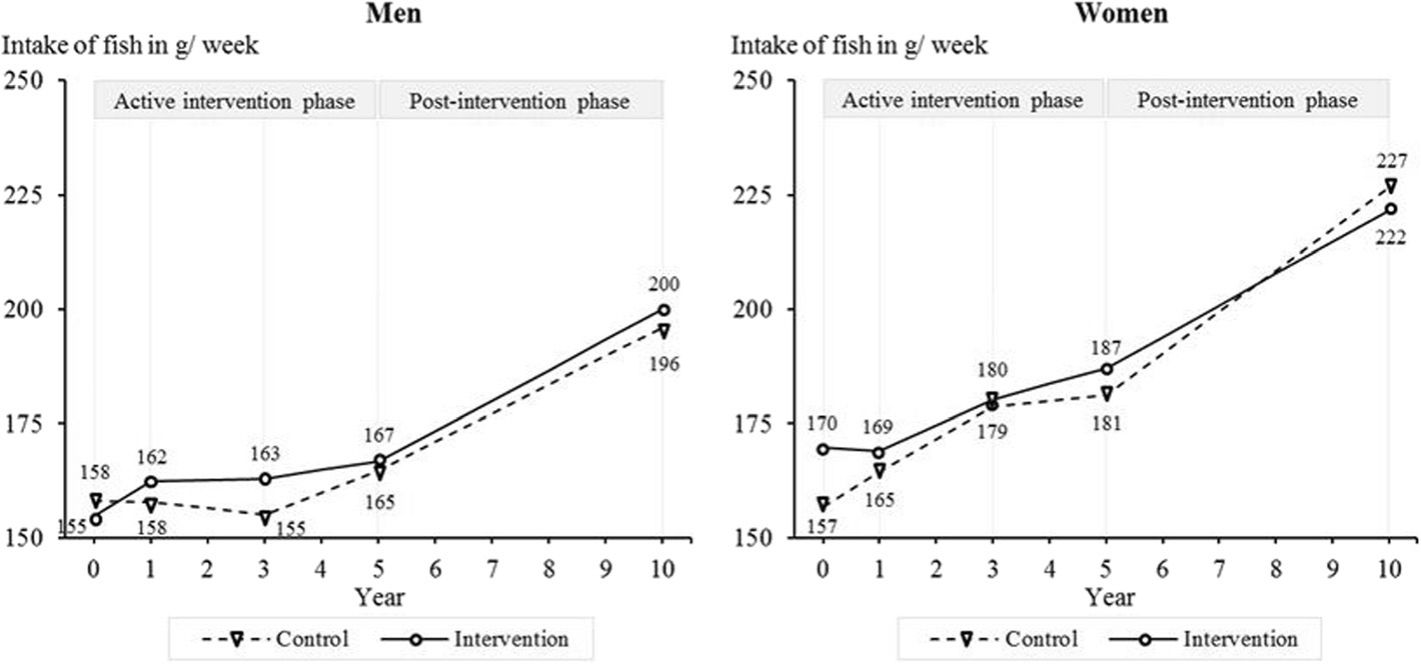
**Intake of fish among women and men from baseline to 10-year follow-up.** *Note:* Results are adjusted for baseline age, living with a partner, education, employment, physical activity, alcohol consumption, smoking, self-rated health, and self-rated risk of IHD associated with dietary habits. Year 0 = baseline.

Men in the intervention and control group did not differ significantly regarding their intake of fish at five years and beyond (10-year follow-up: *M*_Δ_ = 7.3 g/week, 95% CI: -4.0–18.7, *p* = 0.204, *d* = 0.18) (Figure 6). Over ten years, women in the intervention group increased their fish intake less than women in the control group (*M*_Δ_ = -18.1 g/week, 95% CI: -29.1 to -7.2, *p* = 0.001, *d* = -0.33). Since they had a higher intake to start, the fish intake at ten years did not differ significantly between groups (*M* = -5.1 g/week, 95% CI: -14.6–4.4, *p* = 0.294).

## Discussion

A ten-year follow-up of a large randomized intervention study revealed that a healthier lifestyle could be maintained five years after completion of a five-year intervention period. Thus the intervention promoted sustained small-to-medium changes in physical activity and dietary habits. Patterns of long-term changes differed considerably across health behaviors and between men and women.

The most notable changes were obtained regarding the intake of saturated fat. This is hardly surprising given that the intervention placed special emphasis on reducing fat intake, an important recommendation from the national health authorities at that time. From a participant’s perspective, there are many ways to substitute saturated fats with unsaturated fat that can be easily integrated into daily life without notable difference in taste. Further, a slim figure complying with the current ideal of beauty might be an additional motivating factor beyond expected health benefits.

Though somewhat attenuated, the originally achieved beneficial effects of the intervention on physical activity among men remained five years after discontinuation of the intervention. Among women, neither short-term nor long-term effects were found. Findings from previous large-scale intervention trials are inconclusive [9,37]. Lack of time because of work outside the home and domestic work may have inhibited increases in physical activity among women. Alternatively, women in this multi-factorial lifestyle study may have chosen to focus on changing dietary habits instead of physical activity. Besides, gaining more muscle mass and strength might be a motivating factor that is more relevant for men than for women.

Among women, but not men, the effect of the intervention on fruit intake increased over time. Gender-sensitive assessments and programs might be considered to further improve efficacy of lifestyle interventions.

Without continuing intervention, initial improvements in the intake of vegetables and fish declined. Similar to previous trials [5,10], lifestyle in the control group also improved in the longer-term. This is possibly a result of continued assessment that may have made people pay closer attention to their health and lifestyle.

Changes in physical activity and dietary habits found in this study, although significant, were quite small compared to previous trials [38]. However, the small-to-medium effects achieved in this study may be particularly valuable when thinking in terms of population impact. Although the intensity of the intervention was dependent on the individual risk profile, persons from a general population were included in the study regardless of their baseline outcomes and risk status. Thus, this approach may have reached a less selected group of persons compared to previous large-scale long-term trials that have focused on high-risk persons only [11,13,39,40]. However, there was a large selection in participation as persons with low socio-economic status more often declined participation at baseline and also had a higher drop-out rate [41]. Thus, the intervention did not sufficiently reach those with the unhealthiest lifestyle.

It remains important to ascertain why and what specific intervention components were effective. For example, the impact of the quality (e.g., who is delivering the intervention) and intensity of the intervention on its efficacy needs to be considered. Counselors were health professionals trained and supervised in techniques of motivational interviewing. Probably, a more intensive intervention could produce larger and more sustainable improvements in lifestyle. Further, the public health impact might be increased by additionally addressing social and environmental/structural factors.

This paper has some notable limitations. Low participation rates at baseline and follow-up may have led to biased results; especially as we expect that those who have not improved their lifestyle stay away at follow-up. Although improved methods to deal with missing data were used, more successful strategies to approach those currently hard to reach are clearly needed [38]; and adherence to the intervention remains a challenge for future studies [42]. Another limitation concerns the fairly crude assessment of physical activity and dietary habits that may not reflect the complexity of the behaviors. Especially, there could be a gender-specific measurement bias as men and women are not physically active in the same way; women having more home-related practical tasks and men doing more sports. As we relied on self-report only, improvements in lifestyle may partly be explained by social desirability, especially in the intervention group. In addition to that, changes in physical activity and diet cannot be viewed independently of changes in other lifestyle factors. Results concerning the ten-year changes in smoking and alcohol consumption have not yet been published. The synergy that exists between the different lifestyle behaviors needs to be considered for final conclusions.

Very important to have in mind is that, on a population level, when including all 61,301 randomly selected persons, there was no effect of the intervention. Even through more persons in the intervention group achieved healthier diet and physical activity habits in the long-term, the Inter99 study did not reduce incident IHD, stroke or all-cause mortality after ten years [43]. This confirms previous studies showing that screening and lifestyle counseling in a general population is not effective in reducing the burden of IHD on society level [44,45]. The present results show that this lack of effect is not due to attenuation of the changes after end of the lifestyle intervention, but possibly because lifestyle improvements were too small or affected too few or too selected persons to make an influence on IHD on a population level.

Important strengths of this study include proactive recruitment, a priori randomization, and the long follow-up time including a post-intervention phase. Further, this large trial included an individualized and theory-based intervention repeatedly addressing a comprehensive set of health risk factors. The recruitment and intervention strategies used in the Inter99 study have proved to be key elements of successful programs [46-48] that may have the potential to substantially increase the effect on public health [49].

## Conclusion

Systematic screening for high risk in the general population and repeated lifestyle counseling had sustained effects on diet and physical activity five years after its discontinuation – for those who are motivated to participate in an intervention. When developing future lifestyle interventions, a combination of environmental/structural and high-risk strategies should be considered to more successfully reach the whole population regardless of socio-economic factors.

## References

[1] World Health Organization: The European health report 2012: chartering the way to well-being. World Health Organization; 2012.

[2] Lim SS, Vos T, Flaxman AD, Danaei G, Shibuya K, Adair-Rohani H (2012). A comparative risk assessment of burden of disease and injury attributable to 67 risk factors and risk factor clusters in 21 regions, 1990-2010: a systematic analysis for the Global Burden of Disease Study 2010. Lancet.

[3] Ezzati M, Vander Hoorn S, Rodgers A, Lopez AD, Mathers CD, Murray CJ (2003). Estimates of global and regional potential health gains from reducing multiple major risk factors. Lancet.

[4] Marcus BH, Williams DM, Dubbert PM, Sallis JF, King AC, Yancey AK (2006). Physical activity intervention studies: What we know and what we need to know: A scientific statement from the American Heart Association on Nutrition, Physical Activity, and Metabolism (Subcommittee on Physical Activity); Council on Cardiovascular Disease in the Young; and the Interdisciplinary Working Group on Quality of Care and Outcome Research. Circulation.

[5] MacKinnon DP, Elliot DL, Thoemmes F, Kuehl KS, Moe EL, Goldberg L (2010). Long-term effects of a worksite health promotion program for firefighters. Am J Health Behav.

[6] Madden SG, Loeb SJ, Smith CA (2008). An integrative literature review of lifestyle interventions for prevention of type II diabetes mellitus. J Clin Nurs.

[7] Wing RR, Venditti E, Jakicic JM, Polley BA, Lang W (1998). Lifestyle intervention in overweight individuals with a family history of diabetes. Diabetes Care.

[8] Gæde P, Vedel P, Larsen N, Jensen GVH, Parving H-H, Pedersen O (2003). Multifactorial intervention and cardiovascular disease in patients with type 2 diabetes. N Engl J Med.

[9] Knutsen SF, Knutsen R (1991). The Tromsø Survey: the Family Intervention study—the effect of intervention on some coronary risk factors and dietary habits, a 6-year follow-up. Prev Med.

[10] Toobert DJ, Strycker LA, Barrera M, Glasgow RE (2010). Seven-year follow-up of multiple-health-behavior diabetes intervention–HMC. Am J Health Behav.

[11] Cutler JA, Grandits GA, Grimm RH, Thomas HE, Billings JH, Wright NH (1991). Risk factor changes after cessation of intervention in the Multiple Risk Factor Intervention Trial. Prev Med.

[12] Ellingsen I, Hjerkinn EM, Arnesen H, Seljeflot I, Hjermann I, Tonstad S (2006). Follow-up of diet and cardiovascular risk factors 20 years after cessation of intervention in the Oslo Diet and Antismoking Study. Eur J Clin Nutr.

[13] Patterson RE, Prentice RL, Beresford SA, Caan B, Chlebowski RT, Granek I (2004). Dietary adherence in the Women’s Health Initiative Dietary Modification Trial. J Am Diet Assoc.

[14] Lindström J, Ilanne-Parikka P, Peltonen M, Aunola S, Eriksson JG (2006). Sustained reduction in the incidence of type 2 diabetes by lifestyle intervention: follow-up of the Finnish Diabetes Prevention Study. Lancet.

[15] Aadahl M, von Huth SL, Toft U, Pisinger C, Jørgensen T (2011). Does a population-based multifactorial lifestyle intervention increase social inequality in physical activity? The Inter99 study. Br J Sports Med.

[16] Toft U, Kristoffersen L, Ladelund S, Ovesen L, Lau C, Borch-Johnsen K (2008). The impact of a population-based multi-factorial lifestyle intervention on changes in long-term dietary habits. The Inter99 study. Prev Med.

[17] von Huth SL, Ladelund S, Borch-Johnsen K, Jørgensen T (2008). A randomized multifactorial intervention study for prevention of ischaemic heart disease (Inter99): The long-term effect on physical activity. Scand J Public Health.

[18] Jørgensen T, Borch-Johnsen K, Thomsen TF, Ibsen H, Glümer C, Pisinger C (2003). A randomized non-pharmacological intervention study for prevention of ischaemic heart disease: baseline results Inter99. Eur J Cardiovasc Prev Rehabil.

[19] Pisinger C, Vestbo J, Borch-Johnsen K, Jørgensen T (2005). Smoking cessation intervention in a large randomized population-based study. The Inter99 study. Prev Med.

[20] Thomsen T, Davidsen M, Ibsen H, Jørgensen T, Borch-Johnsen K (2001). A new method for CHD prediction and prevention based on regional risk scores and randomized clinical trials; PRECARD and the Copenhagen Risk Score. J Cardiovasc Risk.

[21] Miller WR, Rollnick S (1991). Motivational Interviewing. Preparing people to change addictive behavior.

[22] Janz NK, Becker MH (1984). The Health Belief Model: A decade later. Health Educ Q.

[23] Bandura A (1986). Social foundation of thoughts and action: A social cognitive theory.

[24] Prochaska JO, Velicer WF (1997). The transtheoretical model of health behavior change. Am J of Health Promot.

[25] Toft UN, Kristoffersen LH, Aadahl M, von Huth SL, Pisinger C, Jørgensen T (2006). Diet and exercise intervention in a general population—mediators of participation and adherance: the Inter99 study. Eur J Public Health.

[26] Pisinger C, Vestbo J, Borch-Johnsen K, Thomsen T, Jørgensen T (2005). Acceptance of the smoking cessation intervention in a large population-based study: The Inter99 study. Scand J Public Health.

[27] Haraldsdottir J, Seppänen R, Steinrímsdóttir L, Trygg K, Hagman U (1998). Portion sizes: Nordic standard portions of food and foodstuffs.

[28] Website Inter99 study (Danish) [http://www.inter99.dk]

[29] Saltin B, Grimby G (1968). Physiological analysis of middle-aged and old former athletes: comparison with still active athletes of the same ages. Circulation.

[30] The National Consumer Agency (1995). The Seven Food Guidelines.

[31] Trolle E, Fagt S, Ovesen L (1998). Fruit and vegetables – Guidelines for intake [Frugt og grønt – Anbefalinger for indtagelse]. 244.

[32] Becker W, Konde ÅB, Ohlander EM, Lyhne N, Pedersen AN, Aro A (2004). 4th Edition of the Nordic Nutrition Recommendations – Preface and Chapter 1 and 2.

[33] Toft U, Kristoffersen L, Lau C, Borch-Johnsen K, Jørgensen T (2007). The Dietary Quality Score: validation and association with cardiovascular risk factors: the Inter99 study. Eur J Clin Nutr.

[34] Stata Corp (2013). Stata Statistical Software: Release 13.

[35] Cohen J (1992). A power primer. Psychol Bull.

[36] Little RJ, Rubin DB (2002). Statistical analysis with missing data.

[37] OXCHECK Study Group (1995). Effectiveness of health checks conducted by nurses in primary care: final results of the OXCHECK study. BMJ.

[38] Rees K, Dyakova M, Wilson N, Ward K, Thorogood M, Brunner E (2013). Dietary advice for reducing cardivascular risk. Cochrane Database of Syst Rev.

[39] Eriksson J, Lindström J, Valle T, Aunola S, Hämäläinen H, Ilanne-Parikka P (1999). Prevention of Type II diabetes in subjects with impaired glucose tolerance: the Diabetes Prevention Study (DPS) in Finland. Study design and 1-year interim report on the feasibility of the lifestyle intervention programme. Diabetologia.

[40] Hjermann I, Byre VK, Holme I, Leren P (1981). Effect of diet and smoking intervention on the incidence of coronary heart disease. Report from the Oslo Study Group of a randomized trial in healthy men. Lancet.

[41] Bender AM, Jørgensen T, Helbech B, Linneberg A, Pisinger C (2014). Socioeconomic position and participation in baseline and follow-up visits: the Inter99 study. European Journal of Preventive Cardiology.

[42] Blue CL, Black DR (2005). Synthesis of intervention research to modify physical activity and dietary behaviors. Res Theory Nurs Pract.

[43] Jørgensen T, Jacobsen RK, Toft U, Aadahl M, Glümer C, Pisinger C (2014). Screening and lifestyle counselling in a general population does not reduce ischaemic heart disease: A randomized trial – Inter99. BMJ.

[44] Ebrahim S, Taylor F, Ward K, Beswick A, Burke M, Davey Smith G (2011). Multiple risk factor interventions for primary prevention of coronary heart disease. Cochrane Database Syst Rev.

[45] Krogsbøll LT, Jørgensen KJ, Grønhøj Larsen C, Gøtzsche PC (2012). General health check in adults for reducing morbidity and mortality from disease. Cochrane Database Syst Rev.

[46] Noar SM, Benac CN, Harris MS (2007). Does tailoring matter? Meta-analytic review of tailored print health behavior change interventions. Psychol Bull.

[47] Ory MG, Jordan PJ, Bazzare T (2002). The Behavior Change Consortium: setting the stage for a new century of health behavior-change research. Health Educ Res.

[48] Webb TL, Sniehotta FF, Michie S (2010). Using theories of behaviour change to inform interventions for addictive behaviours. Addiction.

[49] Nigg CR, Allegrante JP, Ory M (2002). Theory comparison and multiple-behavior research: common themes advancing health behavior research. Health Educ Res.

